# The Effect of Nitrogen Fertigation and Harvesting Time on Plant Growth and Chemical Composition of *Centaurea raphanina* subsp. *mixta* (DC.) Runemark

**DOI:** 10.3390/molecules25143175

**Published:** 2020-07-11

**Authors:** Spyridon A. Petropoulos, Ângela Fernandes, Maria Inês Dias, Carla Pereira, Ricardo C. Calhelha, Marija Ivanov, Marina D. Sokovic, Isabel C.F.R. Ferreira, Lillian Barros

**Affiliations:** 1 Department of Agriculture Crop Production and Rural Environment, University of Thessaly, Fytokou Street, N. Ionia, 38446 Magnissia, Greece; 2 Instituto Politécnico de Bragança, Campus de Santa Apolónia, Centro de Investigação de Montanha (CIMO), 5300-253 Bragança, Portugal; afeitor@ipb.pt (Â.F.); maria.ines@ipb.pt (M.I.D.); carlap@ipb.pt (C.P.); calhelha@ipb.pt (R.C.C.); iferreira@ipb.pt (I.C.F.R.F.); 3 Institute for Biological Research “Siniša Stanković”-National Institute of Republic of Serbia, University of Belgrade, Bulevar despota Stefana 142, 11000 Belgrade, Serbia; marija.smiljkovic@ibiss.bg.ac.rs (M.I.); mris@ibiss.bg.ac.rs (M.D.S.)

**Keywords:** antimicrobial activities, antioxidant activity, cytotoxic effects, nitrogen fertilization, organic acids, phenolic compounds, wild edible greens

## Abstract

The aim of the present study was to evaluate the effect of nitrogen fertigation (0, 200, 400, and 600 ppm of total nitrogen) and harvesting time (9 March 2018 and 19 April 2018) on the plant growth, chemical composition, and bioactive properties of *Centaurea raphanina* subsp. *mixta* plants. The highest yield of fresh leaves was observed for the treatment of 200 ppm of N without compromising nutritional value. The increasing nitrogen levels resulted in an increase of α- and total tocopherols and sugars content, especially in the second harvest for tocopherols and in the first harvest for sugars. Similarly, total organic acids and oxalic acid content increased with increasing nitrogen levels in both harvests, while fatty acids composition had a varied response to the tested factors. Pinocembrin neohesperidoside and pinocembrin acetyl neohesperidoside isomer II were the most abundant phenolic compounds with the highest content being observed in the control treatment of the first and second harvest, respectively. The highest antioxidant activity was observed for the control and the 600 ppm treatments of the second harvest for the OxHLIA and TBARS assays, respectively, probably due to the high content of pinocembrin acetyl neohesperidoside isomer II and α-tocopherol, respectively. Finally, cytotoxic effects and antimicrobial properties showed a varied response depending on the treatment. In conclusion, *C. raphanina* subsp. *mixta* has low requirements of nitrogen to achieve the highest yield, while a varied response to the tested fertigation treatments and harvesting time was observed in terms of the chemical composition and the bioactive properties.

## 1. Introduction

The Mediterranean basin is abounding with numerous wild edible species, which have been widely used for food purposes throughout the centuries as part of the traditional Mediterranean diet [1,2,3,4,5]. The increasing number of scientific reports about the beneficial health effects that stem from the regular consumption of such species [6,7,8] has encouraged many research efforts regarding the commercial cultivation of these species, aiming to increase our knowledge on the best agronomic practices [9,10,11,12]. The growing tendency of consumers to a more “natural” lifestyle, including new food habits or shifting to traditional diets, and the well-confirmed health benefits attributed to wild edible species has created market niches and rekindled the interest of the food industry sector [13,14,15,16,17]. Several studies have suggested the cultivation of various wild leafy species in soilless cultivation, aiming to increase productivity through short growth cycles and better nutrient availability, as well as to improve quality of the final products [9,10,18,19,20].

Considering the wild nature of these species, it is of major importance to establish cultivation protocols that will allow the best agronomic performance under diverse conditions and consequently increase product availability and affordability for consumers. However, apart from the achievement of maximum yield, another aspect to be considered is the effect that specific agronomic practices may have on the quality of the edible products, especially in regards to the content of antinutritional factors such as nitrates and oxalic acid, which may accumulate under commercial cultivation conditions [21,22,23]. The bioactive compounds content is also pivotal since all these species are highly appreciated as functional and healthy foods due its high content in phytochemicals. According to the literature, commercial cultivation conditions may negatively affect the quality of edible greens compared to wild relatives by decreasing the beneficial compounds content and/or increasing toxic and antinutritional factors accumulation [24,25,26]. 

Nitrogen is an essential macronutrient that has to be replenished with fertilizer application before and during the growing period based on the cropping schedule of farmers [27,28]. In contrast to conventional crops, several studies have indicated the low requirements of wild or semi-domesticated species in nitrogen and agrochemical inputs, which further emphasize the importance of integrating such novel/alternative species in modern farming systems aiming at sustainable production of horticultural products [9,29,30,31]. However, apart from the total applied amounts, the source of nitrogen also has to be considered for achieving high yields and final products of improved quality and high added value [32,33,34]. The nitrate/ammonium nitrogen ratio is proven to be an effective means to regulate the qualitative traits of leafy greens, since according to the literature feeding plants with nutrient solutions containing higher amounts of ammonium than nitrate nitrogen may decrease oxalic acid content [34,35,36,37,38], while other quality parameters such as tocopherols, phenolic compounds and fatty acids composition, and antioxidant activity could be also affected [32,36].

*Centaurea raphanina* subsp. *mixta* (Asteraceae family) is an underutilized wild edible species of the Mediterranean basin, highly appreciated by rural communities for its tender and bitter edible leaves [22], while recent reports revealed significant bioactive properties, such antimicrobial, antioxidant, and cytotoxic activities associated with its rich content in phytochemicals [22,29,39,40]. So far, scarce literature reports exist regarding the cultivation of the species, while a recent study by our research team indicated its potential use in mildly salt-affected soils [41]. Considering the lack of knowledge regarding agronomic practices for wild and/or semi-domesticated edible species, the aim of the present study was to evaluate the effect of nitrogen application on the yield and quality of *C. raphanina* subsp. *mixta* plants and further suggest the best fertilization practices for the species. For this purpose, four nitrogen levels with different total nitrogen amounts were applied in pot cultivated plants; and plant growth parameters, chemical composition, and bioactive properties of edible leaves were determined. The findings of this study will be very useful in the introduction of the species as a new alternative crop that could be integrated in Mediterranean farming systems and substitute/complement conventional crops that usually require high agrochemical inputs. 

## 2. Results and Discussion

The results regarding the effect of nitrogen fertilization on plant growth are presented in Table 1. The early anthesis of plants did not allow to measure growth parameters in the second harvest; therefore, only the results of the first harvest are presented. The application of nitrogen through irrigation water increased plant fresh weight compared to the control treatment (no nitrogen added), except for the case of 400 ppm, where no significant differences where observed. Moreover, the lowest nitrogen level (200 ppm) resulted in the highest fresh weight compared to the rest of the fertilization treatments, a finding which indicates that the species does not require high inputs of nitrogen fertilizers to achieve their full yield potential. Literature reports for other wild or semi-domesticated species have reported similar preferences for low nitrogen availability [23,29]. Similar results have been suggested for conventional leafy vegetables such as lettuce [42] and spinach [43], where limited availability of nitrogen resulted in a quality improvement without compromising total yield. Regarding the rest of the studied growth parameters, no significant differences were observed between fertilizer treatments, including the control treatment where no nitrogen was added. To the best of our knowledge, this is the first report regarding the fertilizer requirement of the species and the preliminary results of the present study could be a useful basis for the introduction of *C. raphanina* subsp. *mixta* as an alternative/complementary crop for the production of high added horticultural products. In addition, the low requirements in nitrogen fertilizers of the species make it a good candidate for commercial cultivation in sustainable or organic farming systems where nitrogen inputs are limited. 

Wild edible greens are considered very nutritious and are widely used in the Mediterranean diet. The nutritional and energetic value of the studied samples are presented in Table 2. Moisture content was the highest for the control and the 600 ppm treatments of the second harvest, whereas the lowest moisture content was observed in the 600 ppm treatment of the first harvest. The highest and lowest fat content was observed for the 400 ppm and 200 ppm treatments of the second harvest, respectively, while protein content increased with increasing nitrogen levels in both harvests. Ash content was the highest for the control and the 200 ppm treatments of the first harvest, while carbohydrate content was also the highest for the control treatment of the same harvest. Finally, the increased levels of nitrogen (600 ppm) at the first harvest resulted in the highest energetic value of leaves. The present results are within the same range of previous studies reported by our team where the comparison of wild and cultivated plants [22] and the effect of salinity conditions [41] were studied. Moreover, similar studies with other wild edible greens have also suggested a varied response to different crop management practices [44,45,46]. Moreover, considering that fresh weight did not increase by the increased application of nitrogen (Table 1), low levels of nitrogen (200 ppm) may result in high yields without compromising the quality of the final product, with only exception in nitrogen content, which was the highest when plants were fertigated with the 600 ppm solution.

Various wild or semi-domesticated greens are rich sources of tocopherols, while in most cases α-tocopherols is the main detected vitamin E isoform. The individual and total tocopherols’ content is presented in Table 3. The only tocopherols detected in all the tested samples were α- and γ-tocopherols, a finding which is in agreement with our previous reports on the species [22,41]. A variable response to fertigation level and harvesting time was observed, with α-tocopherol being the highest at the second harvest for plants that received 200 or 600 ppm, whereas γ-tocopherol was the highest for the treatment of 400 ppm of the first harvest. Similarly, total tocopherols content was the highest at the second harvest for all treatments where nitrogen was applied. According to Hussain et al. [47], nitrogen rate may affect tocopherols’ content, while nitrogen source also has an impact on tocopherols composition and content [33]. It has to be mentioned that in our study nitrogen fertilization increased the content of individual and total tocopherols compared not only with the control treatment of this study but also with wild plants, which were studied in our previous report [22] Therefore, growing the species with commercial cultivation practices may increase the content of compounds with antioxidant properties, such as tocopherols, while at the same time increase the yield and availability of the product. Moreover, the fact that α- and total tocopherols was higher in the second harvest where leaves are not edible due to a hard texture could create new market niches for nutraceutical uses of the species. Regarding the effect of harvesting time on tocopherols content, similar trends were also observed by Petropoulos et al. [46,48] and these findings can be explained by the high temperatures intervened between the two harvestings. Another factor that could result in increased tocopherols content could be the abiotic stress that plants were subjected to when they received high nitrogen levels, especially when considering that the species is not domesticated and it was cultivated under different conditions from its natural environment [49,50,51]. This explanation is further supported by our previous report where cultivated plants had a higher tocopherols’ content than wild plants. In this respect, further studies are needed where genotypes (ecotypes) from variable environments will be studied in order to unravel the mechanisms responsible for tocopherols content increase and make use of this knowledge for the production of high value added products. 

As seen in Table 4, fructose, glucose, sucrose, and trehalose were detected in all the studied samples in varied amounts depending on the treatment. For example, trehalose was detected in the highest amounts (0.393 g/100 fw) in the first harvest and when plants received 600 ppm of nitrogen through fertigation, whereas fructose was the most abundant sugar (0.27 g/100 g fw) in the second harvest and the plants of the same nitrogen level (600 ppm of nitrogen). Similar findings have been reported by Petropoulos et al. [41] for *C. raphanina* subsp. *mixta* plants grown under saline conditions where the abovementioned sugars were detected within the same range of concentration. Moreover, considering the significance of sugars content in plant protection mechanisms against abiotic stressors either through osmoprotection or via the biosynthesis of protective secondary metabolites [23,52], our results further justify the stressful effect of the growing conditions, which were far different from those of the wild plants [41]. Regarding the effect of harvesting time, plants of the control treatment of the second harvest had the lowest content of individual and total sugars probably due to prolonged lack of nitrogen availability, while nitrogen application resulted in an increase of total sugars content in both harvests and especially in the second one where a 2.6–2.9 fold increase was observed compared to the control treatment. In contrast to our study, Petropoulos et al. [41] reported that total sugars content increased with plant development in *C. raphanina* subsp. *mixta* plants grown under saline conditions, a trend that was observed only for the 200 ppm treatment. Apart from harvest time per se, the practice of successive harvesting has also an impact, since according to Petropoulos et al. [45] and Poli et al. [53], a significant variation in sugars content and composition may be observed from harvest to harvest during the growing period. However, apart from the variable response to the tested levels, it could be suggested that nitrogen application has a positive effect on sugar composition and content and could be used as a cost-effective cultivation practice to regulate the taste of the final product.

Organic acids composition in edible greens is very important especially regarding oxalic acid content, which is considered an anti-nutritional factor. The main detected organic acids in all the studied samples were oxalic acid, followed by citric and malic acid, while ascorbic and fumaric acid were found in lower amounts or in traces (Table 5). These findings are in accordance with previous reports for the species [22,41] and further highlight the increased oxalic acid content of the species when plants are cultivated under commercial conditions. It is already well-established that nitrogen fertilization may increase oxalic acid content, which is formed during the process of nitrogen assimilation by plants as a means to neutralize the released OH^−^ in the case that nitrogen is supplied in nitrate form, or to increase cytoplasm pH and keep the anion-cation balance in place in the case of ammonium nitrogen [38,54]. Therefore, further studies are needed to fine tune the fertilization regime by testing not only the total amount but also the form of applied nitrogen aiming to regulate oxalic acid content [35]. According to Liu et al. [37], there is strong evidence that ammonium nitrogen inhibits nitrate uptake from roots, either through the increased efflux of nitrates or through the down-regulation of specific genes involved in the assimilation of nitrates, which both result in the reduced accumulation of oxalates. As already reported, several wild edible greens could be considered as rich oxalic acid sources and despite the low contribution of these food sources in the overall dietary oxalates intake on a daily basis, special consideration is needed before introducing these species to the market [55,56,57]. Moreover, similar to previous studies, total organic acids content was lower in the second study due to the early flower initiation and allocation of plant metabolites [58], a finding which is more profound in the case of the control treatment of the second harvest where plants were grown under a prolonged nitrogen deprivation compared to the first harvest. 

The lipidic fraction in edible greens is usually low; however, information about fatty acids composition is essential, especially regarding the n-3 fatty acids content and the values of PUFA (Polyunasaturated fatty acids)/SFA (saturated fatty acids) and n-6/n-3 fatty acids ratios. Fatty acids composition is presented in Table 6 with 18 different fatty acids, which have been already reported in *Centaurea raphanina* spp. *mixta* leaves by our team [22,41]. The most abundant compounds were α-linolenic, linoleic, and palmitic acid in percentages that differed among the applied nitrogen levels and harvest times and with no specific trends being observed, except for the increased percentages of palmitic and linoleic acid at the highest nitrogen level (600 ppm) of the second harvest. Similarly, α-linolenic acid percentage was the highest in the control treatment of both harvests and in the treatment of 200 ppm of the second harvest. The rest of the fatty acids (12 out of 18 compounds) were detected at the highest percentage in the control treatment of the second harvest, whereas α-linolenic acid content was the lowest for the same treatment. PUFAs were the most abundant class due to abundance of α-linolenic and linoleic acids (the b values ranged between 39.8–47.2% and 23.72–26.6%, respectively), followed by SFAs represented mostly by palmitic acid (17.8–23.2%). This fatty acids profile is typical in various wild edible greens, which are a good source of polyunsaturated fatty acids with are highly associated with significant beneficial health effects [31,46,48,59,60,61]. Moreover, PUFA/SFA and n6/n3 ratios were within the recommended range that suggests good nutritional value [22,62], regardless of the nitrogen level and time of harvest. This result further supports our previous findings that suggested that *C. raphanina* subsp. *mixta* plants can be grown under commercial cultivation conditions to obtain higher yields without compromising the quality of the final product. 

Wild greens are highly appreciated for their high content in bioactive compounds, which are associated with beneficial health effects. The chromatographic data of phenolic compounds’ identification and quantification of individual and total phenolic compounds are presented in Table 7 and Table 8, respectively. The identified compounds have been previously described by our team [22,41] and classified in the groups of flavonols (peaks 1–4 and 7), flavones (peaks 5 and 6), and *O*-glycosylated flavanones (peaks 8–12). The latter group of compounds was the most abundant one, accounting for 84.0–85.4% and 90.0–90.3% of total phenolic compounds in the first and second harvest, respectively. Nitrogen application had a variable effect depending on the harvesting; thus, the highest total flavonols and total flavones content was observed at the control and 200 ppm treatments of the first harvest, respectively, while the highest total flavanones and total phenolic compounds was recorded for the 400 ppm of the second harvest. Moreover, the increase of nitrogen levels resulted in a decrease for all the groups of compounds in the first harvest compared to the control treatment, whereas a contrasting trend was observed in the second harvest where total phenolic compounds increased mostly due to the increase of flavanones content. This finding could be associated with the higher temperatures and stressful conditions of the second harvest, to the successive harvesting practice and gradual development of plants, as is already mentioned in the literature [29,45,46,48], although the prolonged nitrogen deprivation (0 ppm) resulted in a decrease of almost all the compounds, except for the case of peak 12. Therefore, depending on harvesting time, the increased levels of nitrogen should be preferred in the second harvest aiming to obtain leaves for nutraceutical purposes, while for edible leaves, low levels of nitrogen (up to 200 ppm) should be applied in the first harvest in order to achieve high yields of high quality products. Regarding the individual phenolics, the most abundant compounds were pinocembrin neohesperidoside (peak 9) and pinocembrin acetyl neohesperidoside isomer II (peak 12), in the first and second harvest, respectively. The same compounds were also the richest in our previous study where a similar trend in the respond to harvesting was observed, despite the fact that different treatments were applied (different salinity levels compared to different nitrogen levels of the present study) [41]. Moreover, Mikropoulou et al. [39] also reported the presence of pinocembrin and derivatives in water-based decoctions of *Centaurea raphanina* leaves. 

The health benefits of the so-called Mediterranean diet are associated with the antioxidant potential of the various commonly used ingredients such as wild edible greens. The antioxidant activity of *Centaurea raphanina* spp. *mixta* leaves was determined with two different methods, namely OxHLIA and TBARS, where the antihaemolytic activity and lipid peroxidation inhibition of extracts were assayed (Table 9). The highest activity was observed for the control and the 600 ppm treatments of the second harvest for the OxHLIA and TBARS assays, respectively. In regards to the OxHLIA assay, the observed results could be attributed to the high content of pinocembrim acetyl neohesperidoside isomer II, since according to the literature, this flavanone has exhibited significant bioactive properties [39]. Similarly, the high lipid peroxidation inhibition of plants supplied with 600 ppm in the second harvest could be associated with the high content of α-tocopherol, which contributes to the antioxidant mechanisms of plants [51,63,64,65]. In any case, the leaves of the second harvest showed promising results regarding their antioxidant properties and could be suggested for nutraceutical uses.

Recent analytical protocols have allowed the evaluation of toxic effects of edible species against non-tumor and tumor cell lines, providing useful information regarding the safe consumption and bioactive properties of these species. Cytotoxic effects and antitumor activity are presented in Table 10, where none of the tested samples showed toxicity against non-tumor cell lines (porcine liver primary cultures; PLP2). Moreover, cytotoxicity against tumor cell lines showed a varied response depending on the nitrogen level and harvest time. In particular, the most effective extract against cervical carcinoma cell lines (HeLa) were those obtained from plants of the control treatment on the firs harvest, while extracts from plants of the second harvest and the 200 ppm treatment were most effective against hepatocellular carcinoma cell lines (HepG2). The extracts from plants of the first harvest and the 600 ppm treatment were the most effective against the breast carcinoma (MCF-7) and the non-small cell lung cancer (NCI-H460) cell lines, while the treatment of the 200 ppm of the second harvest was similarly effective against the latter cell line. Similar results were reported by Petropoulos et al. [41] who also observed no toxic effects against non-tumor cell lines and a varied effectiveness against the same tumor cell lines, while Mikropoulou et al. [39] suggested effectiveness against the A5 metastatic spindle and C5N-immortalized keratinocyte cell lines. According to the literature, the various cytotoxic and biological effects observed in *Centaurea* species are correlated with the presence of phytochemicals such as flavonoids sesquiterpene and lactones [66,67,68], which partly could explain the results of our study although no sesquiterpene lactones were detected.

The existing reports have shown significant antimicrobial effects of the extracts of the aerial parts of *Centaurea raphanina* subsp. *mixta*. The antibacterial properties of the tested extracts are presented in Table 11. The effectiveness of all the tested extracts was lower than the streptomycin and ampicillin, which were used as positive controls, while the extracts obtained from the control, 400 ppm and 600 ppm of the first harvest, as well those of the control 400 ppm and 600 ppm of the second harvest had the same minimal inhibition concentration (MIC) and minimal bactericidal concentration (MBC) values against *Staphylococcus aureus*. Moreover, all the extracts were similarly effective against *Escherichia coli* and *Salmonella typhimurium*, while the control, 200 ppm, and 600 ppm treatments of both harvests were more effective against *Bacillus cereus*. Finally, the treatments of 400 ppm (1st harvest) and 200 ppm (2nd harvest) were the most effective against *Listeria monocytogenes* and *Enterobacter cloacae*, respectively. Similar to our study, Petropoulos et al. [22,41] reported lower activity of *C. raphanina* subsp. *mixta* leaves extracts than the same positive controls, while they observed high effectiveness against *Staphylococcus aureus*, *Bacillus cereus* and *Escherichia coli*. The antibacterial properties of *Centaurea* species have been associated with the presence of sesquiterpene lactones [69,70,71], while in our study the abundance of pinocembrin and derivatives could also be correlated with such activities. 

The antifungal activities of the tested extracts are presented in Table 12. In most cases, the tested extracts had higher MIC and minimal fungicidal concentration (MFC) values than the positive controls (bifonazole and ketoconazole), except for the case of *Penicillium funicolosum* and *Trichoderma viride* where the leaves’ extracts of specific treatments were more effective. In particular, the extracts of the control and 400 ppm of the second harvest had lower MIC and similar to bifonazole MFC values against *P. funicolosum*, while the extracts of the control, 400 ppm, and 600 ppm of the first harvest and the control, 200 ppm, and 400 ppm of the second harvest showed lower MIC values against *T. viride.* The response of the tested extracts against the rest of the fungi varied depending on the nitrogen level and the harvest time. The effectiveness against *T. viride* has been also reported in our previous studies [22,41], while Panagouleas et al. [40] suggested that cnicin was responsible for the effectiveness of *C. raphanina* subsp. *mixta* extracts against several fungi with MIC and MFC values lower than those of miconazole. Moreover, Panagouleas et al. [40] and Mikropoulou et al. [39] suggested that flavonoids such as pinocembrin could also exert bioactive properties, a finding which is in accordance with our results.

## 3. Materials and Methods

### 3.1. Plant Material and Experimental Conditions

The experiment was performed in an unheated plastic greenhouse at the University of Thessaly in Volos, Greece. Plant material has been described in detail in previous studies by our team [22,41]. In brief, plants were propagated from seeds collected from wild plants and young seedlings were transferred to 2 L plastic pots containing peat (Klassman-Deilmann KTS2) and perlite (2:1; *v*/*v*). Seeds were sown in seed trays on October 5, 2017 while transplantation took place on January 11, 2018 (one plant per pot). After transplantation, the plants were irrigated and one week after plant establishment, the application of fertilizers started. Four different fertilization treatments were applied, including control where no fertilizer was added (0 ppm of nitrogen). The rest of the nutrient solutions (200, 400, and 600 ppm of nitrogen) were prepared by adding the same amount of a water soluble synthetic fertilizer (Atlas 20-20-20 + TE) in order to have 200 ppm of N-P-K in all the treatments [41], while for the treatments of 400 and 600 ppm, the rest of the nitrogen (200 and 400 ppm, respectively) was added in the form of ammonium nitrate (34.5-0-0; N-P-K). Fifteen pots were used for each treatment (60 pots in total) and arranged according to the completely randomized design (CRD). Fertigation was carried out in regular intervals and depending on growing conditions (once a week at the beginning of the experiment and every two days at the end of the growing period). The amount of nutrient solution added with each fertigation also varied between 50 (early growth stages) and 300 mL (late growth stages). The growing conditions are described in detail by the authors [41].

Plants were harvested two times, namely on March 9, 2018 and April 19, 2018 (first and second harvest, respectively) and when plants achieved a marketable size [45]. Plant material of the first harvest was used for the determination of plant growth according to the methodology described by the authors [22,41]. Due to early anthesis, plant material of the second harvest was only used for chemical composition analysis and bioactivity assays [41]. Samples of fresh leaves were stored at −80 °C, lyophilized, and ground to powder before the chemical analyses.

### 3.2. Chemical Analyses

#### 3.2.1. Proximate Composition and Energetic Value

According to the AOAC methods [72], the proximate composition was determined in the lyophilized plant material and expressed in g/100g of fresh weight (fw). The incineration at 550 ± 15 °C was used to determine the ash content. Crude protein was estimated by the macro-Kjeldahl method (N × 6.25) using an automatic distillation and titration unit (model Pro-Nitro-A, JP Selecta, Barcelona). Soxhlet extraction was used to determine the crude fat, with petroleum ether during 7 h. Total carbohydrates content was calculated by difference using the formula: Total carbohydrates (g/100 g fw) = 100 − (g moisture + g fat + g ash + g proteins). The energetic value was calculated according to the Atwater system using the formula: Energy (kcal/100 g fw) = 4 × (g proteins + g carbohydrates) + 9 × (g fat).

#### 3.2.2. Tocopherols

The extraction of tocopherols from the lyophilized plant material was carried out following the procedure described by Silva et al. [73].The analysis was made using a high performance liquid chromatography coupled to a fluorescence detector (HPLC-FL; Knauer, Smartline system 1000, Berlin, Germany) as described by the authors. For the identification of the tocopherol compounds, chromatographic comparisons with authentic standards were made, and the quantification was performed using tocol as internal standard (IS) and calibration curves obtained from commercial standards (Sigma, St. Louis, MO, USA). The results were expressed in mg per 100 g of fw.

#### 3.2.3. Sugars

The extraction of free sugars from the lyophilized plant material was carried out according to Silva et al. [73]. The compounds were identified using a HPLC with a refraction index detector (HPLC-RI) operating as previously described for the authors cited. Peaks identification was performed by comparisons of their relative retention time (Rt) with authentic standards. Quantification was completed using melezitose as IS, (Sigma-Aldrich, St. Louis, MO, USA). Results were processed in a Clarity Software (Data Apex, Prague, Czech Republic) and expressed in g per 100 g of fw.

#### 3.2.4. Organic Acids

The extraction of organic acids from the lyophilized plant material was done as previously described and optimized by Barros et al. [74] and subsequent determination by ultra-fast liquid chromatography coupled to diode-array detection (UFLC-DAD); (Shimadzu Corporation, Kyoto, Japan). Compounds were identified by comparison of spectra and retention time with the authentic standards (Sigma-Aldrich, St. Louis, MO, USA). The quantification was performed based on calibration curves (245 nm for ascorbic acid and 215 nm for remaining acids). The results were recorded and processed using LabSolutions Multi LC-PDA software (Shimadzu Corporation, Kyoto, Japan), and expressed in mg/100 g of fw.

#### 3.2.5. Fatty Acids

The fatty acids in the lyophilized plant material were determined after the trans-esterification process, as previously described by Silva et al. [73]. The analysis was made using a gas chromatographer DANI model GC 1000 instrument equipped with a split/splitless injector and a flame ionization detector (GC-FID, 260 °C). The identification and quantification of the compound were performed by comparing the relative retention times of fatty acid methyl ester (FAME) from commercial standards. Fatty acids were processed using Clarity Software (DataApex 4.0, Prague, Czech Republic) and the results expressed in percentage.

#### 3.2.6. Phenolic Compounds

Powder from freeze-dried plants was extracted by stirring with 30 mL of ethanol/water (80:20, *v*/*v*) for 1 h and subsequently filtering through Whatman No. 4 paper. The residue was then extracted with an additional 30 mL of ethanol/water for 1 h. The combined hydroethanolic extracts were evaporated to dryness and redissolved in ethanol/water for the evaluation of the phenolic compounds and bioactive assays [75].

For phenolic compounds was weighted 20 mg of prepared lyophilized extracts, re-dissolved in 2 mL of ethanol:water (80:20, *v*/*v*). The compounds were evaluated using a Dionex Ultimate 3000 UPLC system and a diode array coupled in-series to an electrospray ionization mass spectrometry detector (LC-DAD-ESI/MSn) [76]. The identification of the individual phenolic compounds was performed by comparing the retention times, UV-visible spectra, and MS fragmentation patterns of the detected compounds with those of authentic standards; data available from the literature were also used. The quantification was based on the calibration curves of authentic standards. The results were presented as mg/g of plant fw.

### 3.3. Antioxidant Activity

Antioxidant activity was evaluated by applying two cell-based assays: the oxidative haemolysis (OxHLIA) and the thiobarbituric acid reactive substances (TBARS) formation inhibition assays previously described by Spréa et al. [75] using the above-prepared hydroethanolic extracts. The used positive control was Trolox.

#### 3.3.1. OxHLIA Assay

The antihaemolytic activity was determined by the oxidative haemolysis inhibition assay (OxHLIA). The results were expressed as IC_50_ values, which is the extract concentration (µg/mL) required to inhibit oxidative haemolysis of 50% of the erythrocytes for Δt for 60 min.

#### 3.3.2. TBARS Assay

Brain tissues from Sus scrofa were dissected and homogenized with a Tris-HCl buffer (20 mM, pH 7.4) to obtain a homogenate (1:2; *w*/*v*) of the brain tissue and then centrifuged for 10 min at 3000 *g*. The extract samples (0.2 mL) were incubated at 37 °C for 1 h with the porcine brain supernatant (1:2, *w*/*v*; 0.1 mL), FeSO4 (10 mM; 0.1 mL), and ascorbic acid (0.1 mM; 0.1 mL). Tri-chloroacetic (28% *w*/*v*, 0.5 mL) and thiobarbituric (TBA, 2%, *w*/*v*, 0.38 mL) acids were added and the mixture was heated at 80 °C for 20 min and centrifuged for 5 min at 3000 *g*. The color intensity of the malondialdehyde (MDA)-TBA complex in the supernatant was measured by its absorbance at 532 nm. The results were presented as EC_50_ values, which is the extract concentration (µg/mL) that provides 50% of the antioxidant activity.

### 3.4. Hepatotoxicity and Cytotoxicity Assays

This two assays were performed accordingly a procedure optimized by the authors [77]. The hepatotoxicity was evaluated using the sulforhodamine B assay. Primary cell cultures (PLP2) were prepared from porcine liver and tested with different concentrations of the above-prepared hydroethanolic extracts, ranging from 400 to 6.5 µg/mL. The cytotoxicity of the extracts was also evaluated by the same method but using four human tumor cell lines, namely HeLa (cervical carcinoma), HepG2 (hepatocellular carcinoma), MCF-7 (breast adenocarcinoma), and NCI-H460 (non-small cell lung cancer). In both the hepatotoxicity and cytotoxicity assays, ellipticine was used as the positive control and the results were presented as GI_50_ values (µg/mL), corresponding to the extract concentration

### 3.5. Antimicrobial Properties

Antimicrobial properties were measured in the hydroethanolic extracts prepared above, and Gram-positive bacteria *Staphylococcus aureus* (ATCC 11632), *Bacillus cereus* (food isolate), *Listeria monocytogenes* (NCTC 7973), as well as the Gram-negative bacteria *Escherichia coli* (ATCC 25922), *Salmonella typhimurium* (ATCC 13311), and *Enterobacter cloacae* (ATCC 35030) were used. For the antifungal assays, six micromycetes were used: *Aspergillus fumigatus* (ATCC 9197), *Aspergillus niger* (ATCC 6275), *Aspergillus versicolor* (ATCC 11730), *Penicillium funiculosum* (ATCC 36839), *Trichoderma viride* (IAM 5061), and *Penicillium verrucosum* var. *cyclopium* (food isolate); the microdilution method was used [78]. The results were presented as the concentrations that caused the complete inhibition of the bacterial growth (MIC, minimal inhibition concentration), as well as MBC and MFC values (minimal bactericidal concentration and minimal fungicidal concentration, respectively). The used positive controls were streptomycin, ampicillin, ketoconazole, and bifonazole, whereas the negative control was 5% DMSO.

### 3.6. Statistical Analysis

The growth parameters were evaluated in 15 plants per treatment (*n* = 15), while the harvested material from each treatment was used to prepare three batch samples (*n* = 3) for the described chemical analyses. The chemical composition and bioactivity assays were performed in three different samples for each treatment and all the analyses were performed in triplicate. Normal distribution of raw data was checked with Shapiro–Wilk. Data analysis was performed with Statgraphics 5.1. plus (Statpoint Technologies, Inc., Warrenton, VA, USA) and a two-way ANOVA was implemented to evaluate the effect of nitrogen level and harvesting time on each one of the studied parameters. The statistical analysis showed significant interactions between the tested factors for all the tested parameters; therefore, the means of all the treatments were compared with Tukey’s HSD test (*p* = 0.05).

## 4. Conclusions

The results of our study present new information regarding the response of *Centaurea raphanina* subsp. *mixta* plants to nitrogen fertigation and successive harvesting, which are very important cultivation practices for farmers before suggesting the commercial cultivation of the species as a new alternative crop. The highest yield was achieved at the lowest nitrogen level (200 ppm of total nitrogen) without compromises in the quality of the final product. Moreover, the leaves of the second harvest showed promising bioactive properties that suggest possible alternative uses in the nutraceutical and food industry sectors. The present findings are extremely important for the Mediterranean farming sector, which is based on small-scale farming, providing the farmers with new cropping solutions for the production of high value added products. In conclusion, the low nitrogen requirements (up to 200 ppm of total nitrogen) suggest the commercial cultivation of *C. raphanina* subsp. *mixta* in farming systems with low agrochemical inputs. However, further studies are required with various ecotypes collected from diverse environments in order to capture genotypic variability and improve the agronomic performance and quality of the species. 

## Figures and Tables

**Table 1:** Plant growth parameters of *Centaurea raphanina* subsp. *mixta* plants grown in 2 L pots under greenhouse conditions, in relation to harvesting time (1st harvest) and nitrogen level (0–600 ppm) (mean ± SD; *n* = 15).

Harvest *	Nitrogen Level (ppm)	Fresh Weight (g Per Plant)	Rosette Diameter (cm)	Number of Leaves Per Plant	Leaf Thickness (mm)
1st	0	6.93 ± 0.79c	21.33 ± 1.12a	12.5 ± 1.7a	0.80 ± 0.14a
	200	8.88 ± 0.98a	22.53 ± 1.63a	12.40 ± 1.95a	0.74 ± 0.07a
	400	7.12 ± 0.40bc	22.33 ± 1.89a	12.53 ± 1.66a	0.72 ± 0.09a
	600	7.56 ± 0.51b	21.80 ± 1.77a	13.53 ± 1.84a	0.74 ± 0.11a

* Harvest: first harvest (9 March 2018); results from the second harvest (19 April 2018) are not available due to early anthesis of plants. Different Latin letters in the same column indicate significant differences between the means according to Tukey’s HSD test at *p* = 0.05.

**Table 2:** Nutritional value (g/100 g fw) and energetic value (kcal/100 g fw) of *Centaurea raphanina* subsp. *mixta* plants grown in 2 L pots under greenhouse conditions, in relation to harvesting time (1st and 2nd harvest) and nitrogen level (0–600 ppm) (mean ± SD; *n* = 3).

Harvest *	Nitrogen Level (ppm)	Moisture	Fat	Proteins	Ash	Carbohydrates	Energy
1st	0	88.5 ± 0.8d	0.35 ± 0.01f	3.26 ± 0.01d	1.90 ± 0.01a	6.0 ± 0.1a	40.1 ± 0.1c
	200	88.7 ± 0.5d	0.34 ± 0.01f	3.90 ± 0.04b	1.93 ± 0.09a	5.16 ± 0.03c	39.3 ± 0.3d
	400	88.5 ± 0.1d	0.39 ± 0.01d	3.83 ± 0.01c	1.67 ± 0.04bc	5.6 ± 0.1b	41.1 ± 0.1b
	600	87.7 ± 0.3e	0.46 ± 0.02b	4.98 ± 0.04a	1.7 ± 0.2b	5.1 ± 0.1c	44.6 ± 0.5a
2nd	0	91.5 ± 0.9a	0.37 ± 0.01e	1.19 ± 0.01f	1.32 ± 0.02d	5.6 ± 0.1b	30.7 ± 0.1g
	200	91.0 ± 0.1b	0.264 ± 0.001g	2.57 ± 0.01e	1.57 ± 0.03c	4.58 ± 0.02d	31.0 ± 0.1g
	400	89.4 ± 0.3c	0.48 ± 0.01a	3.24 ± 0.04d	1.76 ± 0.01b	5.13 ± 0.01c	37.8 ± 0.1e
	600	90.6 ± 0.2a	0.45 ± 0.01c	3.88 ± 0.01b	1.57 ± 0.01c	3.55 ± 0.01e	33.8 ± 0.1f

* Harvest: first and second harvest were carried out on 9 March 2018 and 19 April 2018, respectively. Different Latin letters in the same column indicate significant differences between the means according to Tukey’s HSD test at *p* = 0.05.

**Table 3:** Composition in tocopherols (mg/100 g fw) of *Centaurea raphanina* subsp. *mixta* plants grown in 2 L pots under greenhouse conditions, in relation to harvesting time (1st and 2nd harvest) and nitrogen level (0–600 ppm) mean ± SD; *n* = 3).

Harvest *	Nitrogen Level (ppm)	α-Tocopherol	γ-Tocopherol	Total Tocopherols
1st	0	0.175 ± 0.004g	0.046 ± 0.003d	0.230 ± 0.007f
	200	0.211 ± 0.004f	0.056 ± 0.001c	0.270 ± 0.007e
	400	0.514 ± 0.008c	0.110 ± 0.009a	0.63 ± 0.02b
	600	0.448 ± 0.004d	0.071 ± 0.003b	0.52 ± 0.01c
2nd	0	0.298 ± 0.005e	0.037 ± 0.002d	0.340 ± 0.007d
	200	0.700 ± 0.001a	0.041 ± 0.002d	0.740 ± 0.001a
	400	0.681 ± 0.003b	0.062 ± 0.001c	0.750 ± 0.007a
	600	0.70 ± 0.02a	0.047 ± 0.003d	0.75 ± 0.02a

* Harvest: first and second harvest were carried out on 9 March 2018 and 19 April 2018, respectively. Different Latin letters in the same column indicate significant differences between the means according to Tukey’s HSD test at *p* = 0.05.

**Table 4:** Composition in sugar (g/100 g fw) of *Centaurea raphanina* v *mixta* plants grown in 2 L pots under greenhouse conditions, in relation to harvesting time (1st and 2nd harvest) and nitrogen level (0–600 ppm) (mean ± SD; *n* = 3).

Harvest *	Nitrogen Level (ppm)	Fructose	Glucose	Sucrose	Trehalose	Total Sugars
1st	0	0.188 ± 0.001d	0.160 ± 0.001c	0.093 ± 0.001g	0.192 ± 0.001e	0.630 ± 0.001d
	200	0.175 ± 0.009de	0.183 ± 0.008b	0.107 ± 0.004f	0.198 ± 0.003d	0.66 ± 0.02cd
	400	0.13 ± 0.02f	0.229 ± 0.006a	0.173 ± 0.007d	0.262 ± 0.006b	0.79 ± 0.01b
	600	0.14 ± 0.03ef	0.184 ± 0.001b	0.126 ± 0.008e	0.393 ± 0.002a	0.84 ± 0.02a
2nd	0	0.070 ± 0.01g	0.059 ± 0.004g	0.082 ± 0.004h	0.125 ± 0.001g	0.336 ± 0.008e
	200	0.24 ± 0.03bc	0.105 ± 0.007e	0.226 ± 0.005b	0.215 ± 0.002c	0.79 ± 0.04b
	400	0.23 ± 0.06c	0.125 ± 0.005d	0.246 ± 0.002a	0.185 ± 0.002e	0.79 ± 0.07b
	600	0.27 ± 0.01a	0.085 ± 0.002f	0.196 ± 0.007c	0.148 ± 0.003f	0.700 ± 0.005c

* Harvest: first and second harvest were carried out on 9 March 2018 and 19 April 2018, respectively. Different Latin letters in the same column indicate significant differences between the means according to Tukey’s HSD test at *p* = 0.05.

**Table 5:** Composition in organic acids (mg/100 g fw) of *Centaurea raphanina* subsp. *mixta* plants grown in 2 L pots under greenhouse conditions, in relation to harvesting time (1st and 2nd harvest) and nitrogen level (0–600 ppm) (mean ± SD; *n* = 3).

Harvest *	Nitrogen Level (ppm)	Oxalic Acid	Malic Acid	Ascorbic Acid	Citric Acid	Fumaric Acid	Total Organic Acids
1st	0	946.3 ± 0.1f	296.7 ± 0.6f	0.28 ± 0.01b	553 ± 3b	0.010 ± 0.001a	1797 ± 4e
	200	1081 ± 3c	396 ± 3c	0.28 ± 0.02b	502 ± 4d	tr	1980 ± 9c
	400	1114 ± 2b	339 ± 4d	0.34 ± 0.02a	380 ± 4h	tr	1834 ± 10d
	600	1197 ± 4a	250 ± 4g	0.17 ± 0.02c	642 ± 6a	tr	2090 ± 14b
2nd	0	232.9 ± 0.6h	223 ± 3h	0.37 ± 0.02a	413 ± 4g	0.010 ± 0.001a	869 ± 1g
	200	873 ± 6g	461 ± 5b	0.37 ± 0.01a	468 ± 5e	tr	1803 ± 4e
	400	1037 ± 7d	588 ± 7a	0.38 ± 0.04a	525 ± 4c	tr	2150 ± 18a
	600	965 ± 6e	327 ± 3e	0.050 ± 0.005d	451 ± 3f	tr	1744 ± 1f

* Harvest: first and second harvest were carried out on 9 March 2018 and 19 April 2018, respectively. Different Latin letters in the same column indicate significant differences between the means according to Tukey’s HSD test at *p* = 0.05.

**Table 6:** Fatty acids composition (relative %) of *Centaurea raphanina* subsp. *mixta* plants grown in 2 L pots under greenhouse conditions, in relation to harvesting time (1st and 2nd harvest) and nitrogen level (0–600 ppm) (mean ± SD).

	1st Harvest * (Nitrogen Level; ppm)				2nd Harvest (Nitrogen Level; ppm)			
Fatty Acid	0	200	400	600	0	200	400	600
C8:0	0.114 ± 0.007b	0.086 ± 0.004d	0.039 ± 0.004g	0.065 ± 0.005e	0.247 ± 0.001a	0.048 ± 0.001f	0.089 ± 0.003d	0.103 ± 0.005c
C10:0	0.101 ± 0.004b	0.080 ± 0.003c	0.050 ± 0.002e	0.063 ± 0.006d	0.206 ± 0.005a	0.061 ± 0.001d	0.078 ± 0.003c	0.087 ± 0.006c
C11:0	0.38 ± 0.02c	0.249 ± 0.004e	0.195 ± 0.008g	0.24 ± 0.01e	0.422 ± 0.007a	0.214 ± 0.001f	0.28 ± 0.01d	0.40 ± 0.01b
C12:0	0.33 ± 0.02b	0.30 ± 0.02c	0.255 ± 0.009d	0.240 ± 0.002e	0.162 ± 0.001f	0.157 ± 0.002g	0.503 ± 0.009a	0.24 ± 0.02e
C14:0	1.3 ± 0.2h	3.1 ± 0.2e	4.24 ± 0.07c	4.4 ± 0.1b	2.29 ± 0.07g	3.2 ± 0.2d	8.50 ± 0.07a	2.6 ± 0.2f
C14:1	nd	nd	0.101 ± 0.001b	0.100 ± 0.002b	nd	nd	nd	0.123 ± 0.007a
C15:0	0.254 ± 0.009b	0.25 ± 0.01b	0.226 ± 0.005c	0.207 ± 0.005e	0.38 ± 0.04a	0.209 ± 0.001e	0.219 ± 0.005d	0.218 ± 0.001d
C16:0	20.3 ± 1.0d	20.8 ± 0.4c	18.0 ± 0.2g	18.8 ± 0.5f	21.8 ± 0.6b	17.8 ± 0.2h	19.27 ± 0.07e	23.22 ± 0.83a
C17:0	0.24 ± 0.01d	0.25 ± 0.01c	0.22 ± 0.01f	0.228 ± 0.006e	0.63 ± 0.02a	0.25 ± 0.02c	0.24 ± 0.02d	0.264 ± 0.001b
C18:0	1.85 ± 0.05c	2.0 ± 0.1b	1.49 ± 0.03f	1.57 ± 0.07e	3.25 ± 0.07a	1.72 ± 0.08d	1.86 ± 0.02c	1.74 ± 0.07d
C18:1n9c	1.56 ± 0.04d	1.56 ± 0.02d	1.78 ± 0.01c	1.77 ± 0.03c	2.1 ± 0.1a	1.49 ± 0.03e	1.9 ± 0.1b	1.49 ± 0.01e
C18:2n6c	23.72 ± 0.09g	24.8 ± 0.2d	24.64 ± 0.01e	24.8 ± 0.7d	24.97 ± 0.06c	25.4 ± 0.2b	24.54 ± 0.09f	26.6 ± 0.5a
C18:3n3	47 ± 1a	43.9 ± 0.7c	46.94 ± 0.03a	46 ± 1b	39.8 ± 0.6e	47.2 ± 0.4a	40.1 ± 0.3d	40.04 ± 0.02d
C20:0	0.31 ± 0.02e	0.31 ± 0.01e	0.251 ± 0.002f	0.24 ± 0.01g	0.72 ± 0.04a	0.35 ± 0.01d	0.368 ± 0.006c	0.40 ± 0.03b
C21:0	0.086 ± 0.001d	0.067 ± 0.004f	0.061 ± 0.001fg	0.057 ± 0.006g	0.37 ± 0.02a	0.078 ± 0.004e	0.143 ± 0.004c	0.171 ± 0.003b
C22:0	0.66 ± 0.05de	0.70 ± 0.05c	0.517 ± 0.003f	0.49 ± 0.01g	0.99 ± 0.04a	0.596 ± 0.004f	0.63 ± 0.04ef	0.82 ± 0.05b
C23:0	0.212 ± 0.004e	0.216 ± 0.002e	0.200 ± 0.004f	0.195 ± 0.003g	0.50 ± 0.02a	0.251 ± 0.006c	0.222 ± 0.002d	0.307 ± 0.003b
C24:0	1.33 ± 0.05a	1.32 ± 0.04a	0.84 ± 0.06e	0.75 ± 0.05f	1.12 ± 0.01c	1.03 ± 0.04d	1.01 ± 0.02d	1.25 ± 0.09b
SFA	28 ± 1d	29.7 ± 0.5c	26.54 ± 0.04e	27.5 ± 0.3d	33.1 ± 0.6a	25.9 ± 0.3f	33.4 ± 0.1a	31.8 ± 0.5b
MUFA	1.56 ± 0.04d	1.56 ± 0.02d	1.88 ± 0.01b	1.87 ± 0.03b	2.1 ± 0.1a	1.49 ± 0.03e	1.9 ± 0.1b	1.61 ± 0.01c
PUFA	71 ± 1bc	68.7 ± 0.5d	71.58 ± 0.03b	70.6 ± 0.2c	64.8 ± 0.7f	72.6 ± 0.2a	64.7 ± 0.2f	66.6 ± 0.5e
PUFA/SFA	2.53 ± 0.07d	2.3 ± 0.5e	2.68 ± 0.03b	2.6 ± 0.3c	2.0 ± 0.6h	2.8 ± 0.2a	1.9 ± 0.1g	2.1 ± 0.5f
n6/n3	0.5 ± 0.1c	0.6 ± 0.1b	0.52 ± 0.01c	0.5 ± 0.1c	0.63 ± 0.03ab	0.5 ± 0.1c	0.61 ± 0.05ab	0.66 ± 0.05a

nd—not detected; * Harvest: first and second harvest were carried out on 9 March 2018 and 19 April 2018, respectively. Caprylic acid (C8:0); capric acid (C10:0); undecylic acid (C11:0); lauric acid (C12:0); myristic acid (C14:0); myristoleic acid (C14:1); pentadecylic acid (C15:0); palmitic acid (C16:0); margaric acid (C17:0); stearic acid (C18:0); oleic acid (C18:1n9); linoleic acid (C18:2n6c); α-linolenic acid (C18:3n3); arachidic acid (C20:0); heneicosylic acid (C21:0); behenic acid (C22:0); tricosylic acid (C23:0); lignoceric acid (C24:0); SFA: saturated fatty acids; MUFA: monounsaturated fatty acids; PUFA: polyunsaturated fatty acids; n6/n3: omega-6/omega-3 fatty acids. Different Latin letters in the same row indicate significant differences between the means according to Tukey’s HSD test at *p* = 0.05.

**Table 7:** Retention time (Rt), wavelengths of maximum absorption in the visible region (λmax), mass spectral data, and tentative identification of the phenolic compounds present in the hydroethanolic extracts of *Centaurea raphanina* subsp. *mixta* plants grown in 2 L pots under greenhouse conditions.

Peak	Rt (min)	λmax (nm)	[M − H]^−^ (*m*/*z*)	MS^2^ (*m*/*z*)	Tentative Identification
1	14.16	349	493	317 (100)	Myricetin-*O*-glucoside
2	18.1	344	477	301 (100)	Quercetin-3-*O*-glucoside
3	18.63	334	461	285 (100)	Kaempherol-*O*-glucoronide
4	20.4	334	579	285 (100)	Kaempherol-*O*-hexoside-pentoside
5	22.14	334	563	269 (100)	Apigenin-*O*-hexoside-pentoside
6	22.9	334	445	269 (100)	Apigenin-*O*-glucoronide
7	25.44	332	665	621 (100), 285 (45)	Kaempherol-*O*-malonyl-pentoside
8	28.28	286/326	549	429 (12), 297 (14), 279 (5), 255 (41)	Pinocembrin arabirosyl glucoside
9	29.47	286/326	563	443 (12), 401 (5), 297 (21), 255 (58)	Pinocembrin neohesperidoside
10	31.39	288/328	591	549 (30), 429 (20), 297 (15), 279 (5), 255 (32)	Pinocembrin acetylarabirosyl glucoside
11	31.79	285/326	605	563 (12), 545 (5), 443 (30), 401 (10), 255 (40)	Pinocembrin acetyl neohesperidoside isomer I
12	32.14	286/328	605	563 (10), 545 (5), 443 (28), 401 (9), 255 (39)	Pinocembrin acetyl neohesperidoside isomer II

**Table 8:** Quantification (mg/g of plant fw) of the phenolic compounds present in the hydroethanolic extracts of *Centaurea raphanina* subsp. *mixta* plants grown in 2 L pots under greenhouse conditions, in relation to harvesting time (1st and 2nd harvest) and nitrogen level (0–600 ppm) (mean ± SD, *n* = 3).

	1st Harvest * (Nitrogen Level; ppm)				2nd Harvest (Nitrogen Level; ppm)			
Peaks	0	200	400	600	0	200	400	600
1	0.079 ± 0.001b	0.077 ± 0.002c	0.068 ± 0.003d	0.080 ± 0.001a	0.054 ± 0.002h	0.057 ± 0.003f	0.061 ± 0.002e	0.055 ± 0.001g
2	0.026 ± 0.001a	0.022 ± 0.003b	0.017 ± 0.001d	0.020 ± 0.001c	0.013 ± 0.001e	0.012 ± 0.001f	0.013 ± 0.001e	0.012 ± 0.002f
3	0.05 ± 0.001a	0.05 ± 0.003a	0.035 ± 0.002bc	0.037 ± 0.001b	0.024 ± 0.001e	0.028 ± 0.001d	0.033 ± 0.001c	0.026 ± 0.001d
4	0.024 ± 0.001b	0.025 ± 0.001a	0.019 ± 0.001d	0.022 ± 0.001c	0.014 ± 0.001g	0.016 ± 0.003f	0.016 ± 0.001f	0.017 ± 0.001e
5	0.023 ± 0.001b	0.026 ± 0.001a	0.02 ± 0.01d	0.022 ± 0.001c	0.015 ± 0.001f	0.019 ± 0.001de	0.019 ± 0.001de	0.018 ± 0.001e
6	0.024 ± 0.001a	0.025 ± 0.001a	0.019 ± 0.001c	0.021 ± 0.001b	0.013 ± 0.001e	0.016 ± 0.001d	0.016 ± 0.001d	0.016 ± 0.001d
7	0.015 ± 0.001b	0.016 ± 0.002a	0.013 ± 0.001c	0.015 ± 0.001b	0.012 ± 0.001d	0.013 ± 0.002c	0.013 ± 0.002c	0.013 ± 0.002c
8	0.057 ± 0.001b	0.022 ± 0.001f	0.035 ± 0.001d	0.029 ± 0.001e	0.005 ± 0.001g	0.032 ± 0.001e	0.074 ± 0.002a	0.047 ± 0.002c
9	0.84 ± 0.002a	0.91 ± 0.01a	0.72 ± 0.02b	0.86 ± 0.04a	0.29 ± 0.01d	0.68 ± 0.03b	0.62 ± 0.06dc	0.56 ± 0.04c
10	0.087 ± 0.001b	0.032 ± 0.001f	0.05 ± 0.01e	0.033 ± 0.001f	0.073 ± 0.001c	0.062 ± 0.001d	0.104 ± 0.003a	0.073 ± 0.002c
11	0.062 ± 0.001c	0.036 ± 0.002d	0.040 ± 0.001d	0.029 ± 0.001e	0.085 ± 0.001a	0.060 ± 0.002c	0.07 ± 0.01b	0.062 ± 0.004c
12	0.36 ± 0.02d	0.29 ± 0.02e	0.24 ± 0.02ef	0.20 ± 0.01f	0.858 ± 0.004a	0.7 ± 0.1c	0.73 ± 0.04b	0.71 ± 0.04bc
Tfols	0.196 ± 0.004a	0.19 ± 0.01b	0.152 ± 0.004d	0.174 ± 0.002c	0.117 ± 0.001g	0.126 ± 0.003f	0.136 ± 0.003e	0.123 ± 0.001f
Tflavones	0.045 ± 0.001b	0.051 ± 0.001a	0.039 ± 0.005d	0.043 ± 0.001c	0.028 ± 0.001f	0.035 ± 0.001e	0.035 ± 0.001e	0.034 ± 0.001e
Tflavn	1.40 ± 0.02c	1.28 ± 0.03d	1.08 ± 0.04f	1.2 ± 0.1e	1.309 ± 0.003d	1.5 ± 0.1b	1.6 ± 0.1a	1.5 ± 0.1b
TPC	1.64 ± 0.02b	1.52 ± 0.03c	1.3 ± 0.1e	1.4 ± 0.1d	1.454 ± 0.003cd	1.7 ± 0.1b	1.8 ± 0.1a	1.7 ± 0.1b

tr—traces; nd—not detected. Tfols: total flavonols; Tflavones: total flavones; Tflavn: total flavanones; TPC: total phenolic compounds. * Harvest: first and second harvest were carried out on 9 March 2018 and 19 April 2018, respectively. Standard calibration curves used for quantification: apigenin-7-*O*-glucoside (y = 10683x – 45794, R² = 0.996, LOD = 0.10 µg/mL and LOQ = 0.53 µg/mL, peaks 5 and 6); myricetin (y = 23287x − 581708, R² = 0.9988, LOD = 0.23 µg/mL and LOQ = 0.78 µg/mL, peak 1); naringenin (y = 18433x + 78903, R² = 0.9998, LOD = 0.17 µg/mL and LOQ = 0.81 µg/mL, peaks 8, 9, 10, 11, and 12); and quercetin-3-*O*-glucoside (y = 34843x − 160173, R² = 0.9998, LOD = 0.21 µg/mL and LOQ = 0.71 µg/mL, peaks 2, 3, 4, and 7). Different Latin letters in the same row indicate significant differences between the means according to Tukey’s HSD test at *p* = 0.05.

**Table 9:** Antioxidant activity of *Centaurea raphanina* subsp. *mixta* plants grown in 2 L pots under greenhouse conditions, in relation to harvesting time (1st and 2nd harvest) and nitrogen level (0-600 ppm) (mean ± SD; *n* = 3).

Harvest *	Nitrogen Level (ppm)	OxHLIA (IC_50_; µg/mL); Δt = 60 min	TBARS (EC_50_, μg/mL)
1st	0	257 ± 11a	46 ± 1b
	200	172 ± 6c	30 ± 1d
	400	219 ± 6b	48 ± 2b
	600	223 ± 9b	34 ± 2c
2nd	0	34 ± 3g	65 ± 1a
	200	74 ± 4f	28 ± 2e
	400	65 ± 5e	34 ± 1c
	600	105 ± 9c	26.5 ± 0.1f

EC_50_: extract concentration corresponding to a 50% of antioxidant activity. Trolox EC_50_ values: 23 µg/mL (TBARS inhibition) and 19.6 µg/mL (OxHLIA Δt = 60 min). * Harvest: first and second harvest were carried out on 9 March 2018 and 19 April 2018, respectively. Different Latin letters in the same column indicate significant differences between the means according to Tukey’s HSD test at *p* = 0.05.

**Table 10:** Cytotoxicity and antitumor activity (GI_50_ values μg/mL) of *Centaurea raphanina* subsp. *mixta* plants grown in 2 L pots under greenhouse conditions, in relation to harvesting time (1st and 2nd harvest) and nitrogen level (0–600 ppm) (mean ± SD; *n* = 3).

		Cytotoxicity to Non-Tumor Cell Lines	Cytotoxicity to Tumor Cell Lines			
Harvest *	Nitrogen Level (ppm)	PLP2 (porcine Liver Primary Culture)	HeLa (Cervical Carcinoma)	HepG2 (Hepatocellular Carcinoma)	MCF-7 (Breast Carcinoma)	NCI-H460 (non-Small Cell Lung Cancer)
1st	0	>400	343 ± 13a	342 ± 19b	>400	>400
	200	>400	>400	>400	>400	>400
	400	>400	>400	>400	>400	>400
	600	>400	289 ± 17b	>400	320 ± 13	319 ± 1a
2nd	0	>400	>400	>400	>400	>400
	200	>400	>400	354 ± 12a	>400	317 ± 8a
	400	>400	>400	>400	>400	>400
	600	>400	294 ± 14b	>400	>400	>400

GI_50_ values correspond to the sample concentration responsible for 50% inhibition of growth in tumor cells or in a primary culture of liver cells-PLP2. GI_50_ values for Ellipticine (positive control): 1.2 μg/mL (MCF-7), 1.0 μg/mL (NCI-H460), 0.91 μg/mL (HeLa), 1.1 μg/mL (HepG2) and 2.3 μg/mL (PLP2). * Harvest: first and second harvest were carried out on 9 March 2018 and 19 April 2018, respectively. Different Latin letters in the same column indicate significant differences between the means according to Tukey’s HSD test at *p* = 0.05.

**Table 11:** Antibacterial activity (MIC and MBC in mg/mL) of *Centaurea raphanina* subsp. *mixta* plants grown in 2 L pots under greenhouse conditions, in relation to harvesting time (1st and 2nd harvest) and nitrogen level (0–600 ppm) (mean ± SD; *n* = 3).

Harvest *	Nitrogen Level (ppm)	MIC/MBC	*Staphylococcus aureus* (ATCC 11632)	*Bacillus cereus* (Food Isolate)	*Listeria monocytogenes* (NCTC 7973)	*Escherichia* *coli* (ATCC 25922)	*Salmonella typhimurium* (ATCC 13311)	*Enterobacter cloacae* (ATCC 35030)
1st	0	MIC	1	0.5	2	0.5	2	2
		MBC	2	1	4	1	4	4
	200	MIC	2	0.5	2	0.5	2	2
		MBC	4	1	4	1	4	4
	400	MIC	1	1	2	0.5	2	1
		MBC	2	2	4	1	4	2
	600	MIC	1	1	2	0.5	2	2
		MBC	2	2	4	1	4	4
2nd	0	MIC	1	0.5	2	0.5	2	2
		MBC	2	1	4	1	4	4
	200	MIC	1	0.5	1	0.5	2	2
		MBC	2	1	2	1	4	4
	400	MIC	2	1	2	0.5	2	2
		MBC	4	2	4	1	4	4
	600	MIC	1	0.5	2	0.5	2	2
		MBC	2	1	4	1	4	4
	Streptomycin	MIC	0.1	0.025	0.15	0.1	0.1	0.025
		MBC	0.2	0.05	0.3	0.2	0.2	0.05
	Ampicillin	MIC	0.1	0.1	0.15	0.15	0.1	0.1
		MBC	0.15	0.15	0.3	0.2	0.2	0.15

* Harvest: first and second harvest were carried out on 9 March 2018 and 19 April 2018, respectively; MIC—minimal inhibition concentration; MBC—minimal bactericidal concentration.

**Table 12:** Antifungal activity *Centaurea raphanina* (MIC and MFC mg/mL) of *Centaurea raphanina* subsp. *mixta* plants grown in 2 L pots under greenhouse conditions, in relation to harvesting time (1st and 2nd harvest) and nitrogen level (0–600 ppm) (mean ± SD; *n* = 3).

Harvest *	Nitrogen Level (ppm)	MIC/MFC	*Aspergillus fumigatus* (ATCC 9197)	*Aspergillus niger* (ATCC 6275)	*Aspergillus versicolor* (ATCC 11730)	*Penicillium funiculosum* (ATCC 36839	*Trichoderma viride* (IAM 5061)	*Penicillium verrucosum var. cyclopium* (Food Isolate)
1st	0	MIC	0.5	0.25	0.25	0.25	0.12	0.25
		MFC	1	0.5	0.5	0.5	0.25	0.5
	200	MIC	0.5	0.5	0.25	0.25	0.25	0.5
		MFC	1	1	0.5	0.5	0.5	1
	400	MIC	0.5	0.5	0.25	0.25	0.12	0.25
		MFC	1	1	0.5	0.5	0.25	0.5
	600	MIC	0.25	0.25	0.25	0.25	0.12	0.5
		MFC	0.5	0.5	0.5	0.5	0.25	1
2nd	0	MIC	0.5	0.25	0.5	0.12	0.12	0.25
		MFC	1	0.5	1	0.25	0.25	0.5
	200	MIC	1	0.5	0.5	0.25	0.12	0.25
		MFC	2	1	1	0.5	0.25	0.5
	400	MIC	0.5	0.5	0.5	0.12	0.12	0.25
		MFC	1	1	1	0.25	0.25	0.5
	600	MIC	1	0.5	0.5	0.25	0.25	0.25
		MFC	2	1	1	0.5	0.5	0.5
	Bifonazole	MIC	0.15	0.15	0.1	0.2	0.15	0.1
		MFC	0.2	0.2	0.2	0.25	0.2	0.2
	Ketoconazole	MIC	0.2	0.2	0.2	0.2	1	0.2
		MFC	0.5	0.5	0.5	0.5	1.5	0.3

* Harvest: first and second harvest were carried out on 9 March 2018 and 19 April 2018, respectively; MIC—minimal inhibition concentration; MFC—minimal fungicidal concentration.
